# Orthodontic and Maxillofacial Surgery Treatment in Achondroplasia for Orofacial Alterations: A Systematic Review and Preliminary Age‐Stratified Guidelines

**DOI:** 10.1111/ocr.70108

**Published:** 2026-04-06

**Authors:** Marco Farronato, Maria Francesca Bedeschi, Cristina Grippaudo, Gianluca Martino Tartaglia, Berardo Rinaldi, Cinzia Maspero

**Affiliations:** ^1^ Department of Biomedical Surgical and Dental Sciences University of Milan Milan Italy; ^2^ Fondazione IRCCS Cà Granda Ospedale Maggiore Policlinico Milan Italy; ^3^ Clinical Genetics Unit, Fondazione IRCCS Ca' Granda Ospedale Maggiore Policlinico Milan Italy; ^4^ Università Cattolica del Sacro Cuore Rome Italy; ^5^ Fondazione Policlinico Gemelli IRCCS Rome Italy

**Keywords:** achondroplasia, craniofacial displasia, maxillofacial surgery, orthodontics, syndromes, systematic review

## Abstract

This systematic review aimed to collect and appraise the clinical outcomes of all orthopaedic, orthodontic and surgical interventions in ACH patients. Following PROSPERO protocol, multiple database sources were searched to December 2024 with no language restrictions for (i) genetically confirmed ACH; (ii) any orthodontic/orthopaedic/orthognathic treatment. Two reviewers independently screened, extracted data and applied Joanna Briggs Institute appraisal tools. Results were synthesised to collect the evidence and provide available guidelines. Thirteen studies met the criteria (11 case reports, 2 case series) comprising 16 treated individuals (3–23 y.o). Five patients aged 10–16 years achieved stable correction with orthopaedic treatment followed by fixed appliances, without extractions or surgery at ≥ 24‐month follow‐up. Eight post‐growth patients (> 16 years) required either extractions (*n* = 4) or maxillofacial surgery (*n* = 5) to attain satisfactory occlusion; long‐term stability was documented in only two. Two preschool children underwent mid‐face distraction and were successfully decannulated. Risk‐of‐bias was generally moderate. Early orthopaedic‐orthodontic protocols (≤ 16 years) corrected transverse and sagittal discrepancies in ACH, whereas combined orthodontic–surgical approaches were usually indispensable after growth completion. Future prospective are essential to strengthen the evidence base age‐stratified guidelines.

**PROSPERO registration number:** CRD42024582720

## 1 Introduction

Achondroplasia (ACH) is the most prevalent type of disproportionate short stature. The incidence of the condition is estimated to be around 1 in 25,000 and 1 in 30,000 live births worldwide [1]. It is primarily characterised by disproportionate short stature with rhizomelic limb shortness and macrocephaly with a broad forehead, broad and depressed nasal bridge, malar hypoplasia, and brachydactyly with a trident hand. The main associated medical problems are neurological issues (stenosis of the foramen magnum, spinal stenosis), ENT issues (recurrent middle ear infections, hearing loss, OSAS), dental issues, obesity, and psychosocial issues. Achondroplasia (ACH) is an autosomal dominant condition, in 80% of cases caused by de novo mutations in the fibroblast growth factor receptor 3 (FGFR3), specifically a G‐to‐A transition at nucleotide 1138, which results in the substitution of glycine with arginine at codon 380 (G380R) of the FGFR3 protein. This aberrant signalling of FGFR3 inhibits chondrocyte proliferation and differentiation, particularly in the growth plates of long bones, leading to phenotypic features of achondroplasia. There are several other syndromes and diseases that cause up to 50 known different classifications according to ORPHANET [2]. The most of individuals with ACH are diagnosed in the postnatal period, although it may be suspected prenatally by ultrasound evidence of femoral length deflection in the third trimester, macrocephaly by the 24th gestational week. Until a few years ago, the primary treatment for achondroplasia was surgery, particularly limb lengthening, with the goal of improving growth and body proportions. In recent years, new treatment options have emerged that offer significant hope for patients and their families. In particular, Vosoritide (Voxzogo), a synthetic analog of C‐type natriuretic peptide (CNP), approved first by the FDA, and subsequently by EMA and AIFA, was introduced for restoring the balance and addressing the effects of the FGFR3 pathway, that is overactive and suppresses bone growth [3]. The administration is daily through subcutaneous injections, and improves growth velocity and body proportions, with a positive impact on their physical development. The long term effects, the safety and the interaction with other orthopaedic therapies are still unknown as, at the time of writing it has been distributed on a large scale for less than 3 years [4, 5]. For example, the effects on facial bones and especially on the mandibular growth are under investigation [6].

The European consensus outlined a document established by the European Achondroplasia Forum (EAF) for optimal care in managing ACH made by anonymous voting by a committee of experts. The committee established six principles to indicate the importance of lifelong multidisciplinary treatment and their goals while maintaining the patient's autonomy and a dialogue with the family [7, 8].

Clinically, according to a recent survey, the most frequent complications are sleep apnoeas, otitis, stenosis of the foramen magnum, pressure on the spinal cord, and hearing impairments, and typically lengthening surgery is a treatment option [Bedeschi]. However, also pain is often perceived both to knee and shoulder manifesting often a chronic evolution and causing a lower quality of file [9].

Orthodontic treatment plays a pivotal role in the management of ACH and addresses both functional and aesthetic features of the patients. It focuses on addressing the dental and skeleto‐facial discrepancies from the early stages of growth [10, 11, 12]. The mid area of the face is usually affected from underdevelopment of the maxilla relative to the skull base, presenting a hypoplasic development pattern from the nasal suture to the maxillary bone, as a result the mandible might be in third class due to the underdevelopment of the maxilla [13]. It might cause anterior crossbite due to the relative mandibular prognathism. The hypoplasia is attributed to the premature fusion of the cranial base synchondroses, which reduces the growth of the anterior cranial base and consequently affects midfacial development. Patients also often present comorbidity in the same area, with respiratory affection including sleep apnea, both obstructive and central and otitis media, both correlated with the growth pattern of the maxillary area and middle third of the face. This is why palatal expansion has a pivotal role influencing the nasal resistance and enhancing the nasal pyramid morphology in paediatric patients [14, 15, 16, 17].

Orthodontic interventions for ACH must consider the exposure to unique craniofacial characteristics such as midfacial hypoplasia, retained teeth, and agenesis, which differentiate these patients from those with other craniofacial conditions. Correction of skeletal Angle Class III can be treated or compensated during growth while after growth is accomplished by surgical approach, for example LeFort osteotomy [18].

Mori et al. in 2017 described a case where only early orthodontic treatment was performed, without the need of an ortho/surgical approach [19]. The timing of orthodontic intervention is critical being early intervention essential to address crossbites and crowding, yet the craniofacial growth along the development must be considered to avoid long‐term negative outcomes. The outcomes of orthodontic treatment in ACH should aim for improvements not only in occlusion but also the prevention of functional complications or in the airways and achieving facial harmony. A guideline for the orthodontic approach is still lacking, and collecting and updating the current evidence is necessary to increase the therapeutic effectiveness in the short and long term.

The present study aims to systematically review the literature and to collect the evidence on the orthodontic treatment in ACH patients.

## 2 Materials and Methods

### 2.1 Research Question

The research question was elaborated according to the PI(E)CO format for systematic reviews.

#### 2.1.1 PIECO

The aim of this systematic review refers to the PIECO question:

Population: Patients diagnosed with ACH; undergoing orthodontic treatment.

Intervention: Orthodontic or a combination with orthopaedic, functional or surgical treatment treated with orthodontics, orthopaedic, and/or orthognathic surgery; untreated; correlated with the results achieved.

Exposure: The unique craniofacial and dental characteristics associated with achondroplasia such as midfacial hypoplasia, dental crowding, retained teeth, agenesis, relative prominent mandible, skeletal discrepancies and other complications.

Comparison: Similar patients presenting the same complications but without ACH, or untreated patients with ACH.

Outcome: Clinical outcomes, Improvement in occlusion, craniofacial harmony, stability through time, and complications.

What are the clinical evidences guiding the orthodontic treatment in patients with achondroplasia compared to non‐achondroplastic individuals or those with other craniofacial syndromes?

### 2.2 Protocol and Registration

Before starting the review process, we developed a research protocol based on the research questions. This protocol was registered in the PROSPERO database (International Prospective Register of Systematic Reviews) with registration number CRD (CRD42024582720). To ensure transparency, it was designed following the guidelines of the Cochrane Handbook for Systematic Reviews and PRISMA‐P (Preferred Reporting Items for Systematic Review and Meta‐Analysis Protocols). The protocol is accessible at [20]. To avoid duplicating existing work or overlapping with other reviews or meta‐analyses on the same topic, besides the preliminary research on PROSPERO and Cochrane, we conducted a preliminary search in PubMed. Using criteria similar to those outlined in this paper, the search confirmed the absence of a systematic review on the topic.

### 2.3 Information Source

The search strategy was implemented to identify the selected studies from different relevant sources. Electronic databases searched included PubMed, Embase, Scopus, and the Cochrane Library. Additionally, grey literature was systematically explored to capture studies that might not be indexed in traditional bibliographic databases. Sources of grey literature included ProQuest Dissertations & Theses, OpenGrey, and conference proceedings relevant to the topic.

To identify ongoing and recently completed studies, clinical trial registries such as ClinicalTrials.gov and the WHO International Clinical Trials Registry Platform (ICTRP) were also searched. Furthermore, hand‐searching was conducted in key orthodontic journals and reference lists of included studies to identify any additional eligible studies that may have been missed during the database searches.

The searches were conducted without language restrictions, with translation services employed when necessary to include non‐English studies.

### 2.4 Search Strategy

The search strategy used was designed with the help of an expert from the institutional biomedical library. The search terms used were extracted from MeSH (Medical Subject Heading), separated by boolean operators and adapted to every database scanned. The following MeSH resulted in the selection: “Achondroplasia” AND “Orthodontics” OR “Oral Surgical Procedures” AND “Humans”. A detailed document on the research strategy was made available online at the link [21]. All searches were finalised by December 2024, and the results were imported into systematic review management software (Covidence) to manage screening and organisation of the final database. The detailed search strategies for each database, including keywords, MeSH terms, and Boolean operators used, are provided in the Data S1.

The criteria used for inclusion/exclusion were divided into sections: population, treatment and outcomes. For the population included: only human patients of any age with a genetically confirmed diagnosis of achondroplasia (FGFR3 mutation or explicit genetic confirmation reported) presenting craniofacial alterations attributable to their genetic condition. For the intervention: only studies reporting at least one intervention among orthodontic treatment (fixed, removable, interceptive, or comprehensive), orthopaedic and functional treatment (rapid maxillary expansion, facemask) orthognathic and craniofacial surgery or multidisciplinary were considered. The outcomes included skeletal or occlusal changes registered at the beginning and at the end of the treatment.

### 2.5 Study Selection

Two reviewers among the authors were encharged of the study selection (**; **). In case of discrepancies a third senior researcher among the authors was selected to confirm the final decision. The articles were imported in two digital managers Rayyan (Qatar Computing Research Institute, Doha, Qatar) and Endnote (v. X8, Clarivate Analytics, London, UK). The software allowed to exclude possible duplicates and to check the references of each selected manuscript. Due to the initial screening the collected studies did not present consistent final outcomes to support a metanalysis study. Therefore, due to insufficient statistical data metanalysis was not possible.

**FIGURE 1:**
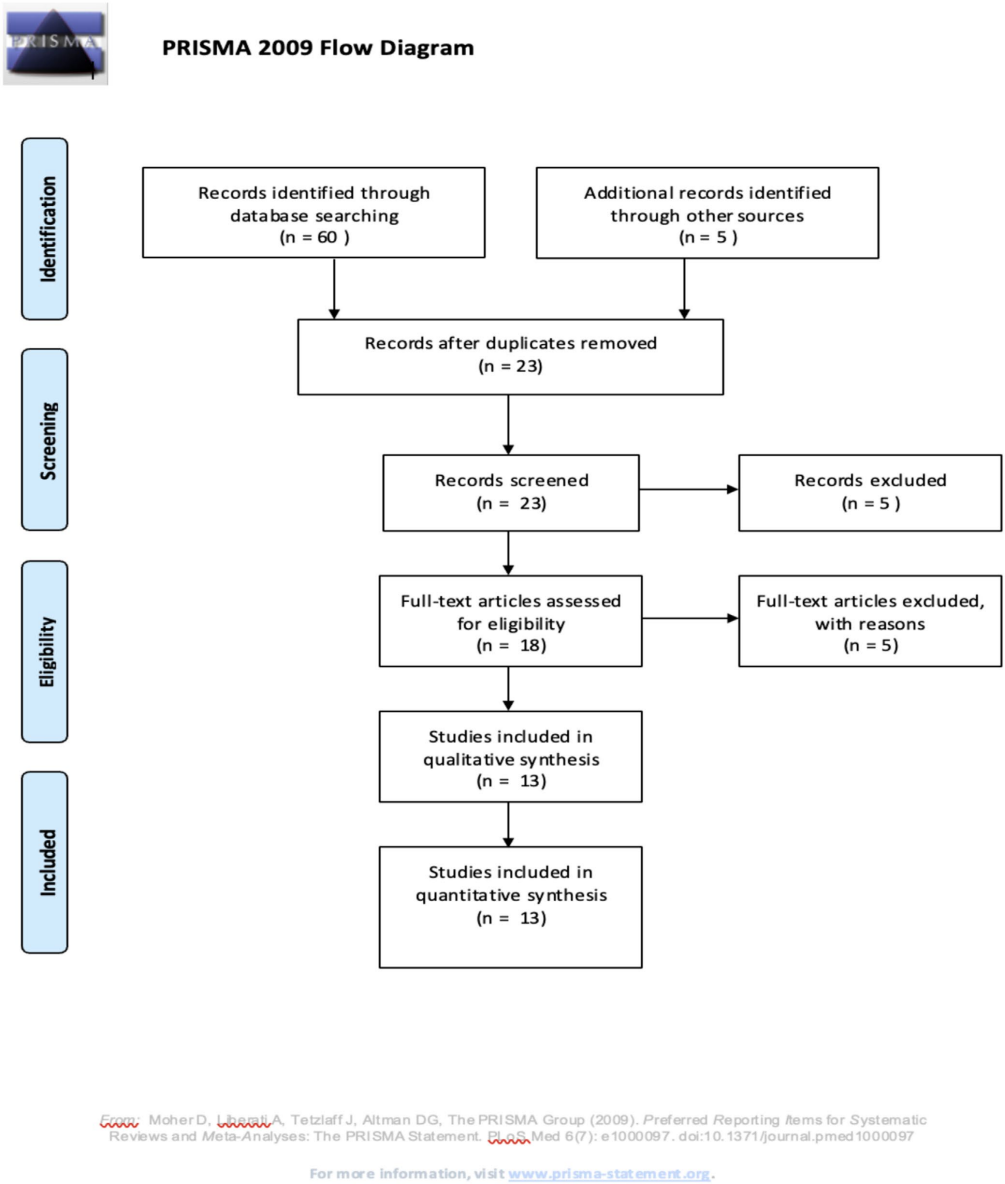
Shows the Prisma diagram of the study.

The articles, after exclusion, were screened according to their Title and Abstract and included or excluded according to the relevance to the topic. In detail any orthodontic treatment in at least one human patient presenting any form, and genetic manifestation of confirmed Achondroplasia was considered. In detail the orthodontic treatment comprised: orthodontic or combined orthodontic‐orthopaedic/orthognathic surgical interventions, reported alone or as part of a multidisciplinary management approach. An overview of the selection process was made in accordance with the PRISMA guidelines and displayed through a diagram (Figure 1).

### 2.6 Data Collection and Synthesis

Once selection the criteria were applied, the final list of included manuscript was edited and the full text of each manuscript was attained through access grande by the library of the institutions of the authors. When the option was not available, each Journal's editorial office was contacted, when unresponsive the authors contacted directly the corresponding authors. The following data was extracted:

Study details: Author(s), year of publication, journal, country, study design.

Population: Number of participants, age range, sex, diagnostic criteria for ACH, relevant comorbidities, pharmaceutical therapy/previous interventions.

Orthodontic diagnosis and skeletal class (prior to the treatment).

Interventions: Type of orthodontic appliance(s) used, adjunctive therapies (e.g., orthopaedic expansion, functional appliances, surgical treatment) divided into: orthopaedic, orthodontic and surgical, duration of the treatment, compliance.

Comparison(s): Control groups (if available) or standard reference values.

Outcomes and key findings: Quantitative or qualitative measures of occlusal changes, facial skeletal alterations, airway assessments, adverse events, outcomes, statistical results, effect sizes (if reported), overall conclusions.

Follow‐up: Duration and intervals of follow‐up, long‐term stability and complications.

The extraction was carried out by the same authors who led the study selection and discrepancies were solve by the intervention of a third expert reviewer among the authors. The domains of data extraction were made a priori according to Cochrane and Prisma recommendations.

### 2.7 Risk of Bias

To assess the quality and reliability of the included studies, two reviewers utilised a standardised tool specific to each study design: Joanna Briggs Institute (JBI) critical appraisal tools for Case Reports and Case Series Adapted checklists [22]. Disagreements were resolved by discussion and by consulting a senior researcher among the authors. The final results were considered upon finalisation, and a high presence of unclear, partial or “no” scores would decrease the final weight of the conclusion taken on the corresponding manuscripts with low scores.

### 2.8 Implications for Practice and Guidelines Proposal

Based on the synthesised evidence, clinical recommendations will be formulated addressing:

Early Interceptive Orthodontics: Ideal timing and types of appliances.

Growth Monitoring: Integration with paediatric endocrinology, especially in patients undergoing GH or vosoritide therapy.

Skeletal Discrepancy Management: Criteria for orthognathic surgery, role of maxillary expansion, and functional appliances in midfacial hypoplasia.

Multidisciplinary Collaboration: Coordination with otolaryngologists, geneticists, and orthopedists to evaluate airway and skeletal development.

The proposed guidelines will be structured to address prenatal consultation, early childhood interventions, adolescent and adult considerations, and long‐term follow‐up.

## 3 Results

### 3.1 Study Selection

According to our research conducted in the selected database, a total of 60 manuscripts were initially retrieved, through database searching (PubMed, Embase, Scopus, Cochrane Library). Additional sources as grey literature, or conference proceedings were also screened. After removing duplicates, 23 studies remained. Following the initial title/abstract screening, 18 full‐text articles were assessed for eligibility; of these, 5 were excluded (reasons included: no orthodontic treatment reported, no confirmed achondroplasia diagnosis, or non‐human study), leaving 13 articles for qualitative synthesis and ROB analysis. A full summary is available on Table 1.

**TABLE 1:** Shows the studies selected and the collected variables from each.

Authors	Patients	Age	Gender	Previous treatment/pharmacological	Orthodontic problem	Intervention	Orthopaedic	Orthodontic	Extraction	Surgery	Duration of the intervention	Comparison	Outcomes	Follow up	Compliance	Field
**Pineau**, Farrow, Nicot, Ferri	3	12;16;11	F/M/M	Male 1: Le Fort III distraction osteogenesis and Orthodontic alignment	Female 1: midface hypoplasia, concave facial soft‐tissue profile, and vertical chin excess. Intraoral assessment showed Class III molar relationship. Male 1: concave profile, Class III molar relationship with anterior open bite. Male 2: skeletal and dental Class III with edge to edge incisor relationship	Female 1: transverse maxillary expansion, alignment, levelling, and lifting of compensations. Surgical Palate expander. Bimaxillary orthognathic surgery was performed with Le Fort I advancement osteotomy, bilateral sagittal split osteotomy, and reduction genioplasty. Male 1: bimaxillary orthognathic surgery with Le Fort I advancement osteotomy and bilateral sagittal split osteotomy. Male 2: maxillary traction with fixed plates. Orthodontic expansion	y;n;y.	y;n;y.	n;n;n.	y;y;n.	F 12 to 21; M 16 to 19; M 11 to 13 (failed)		Female 1: Class I occlusion, profile improvement. Male 1: Class I occlusion, profile improvement. Male 2, failed edge to edge traction correction	n/a		Orthodontics; maxillofacial surgery
**Mori**, Matsumoto, Kawai, Ozawa, Horiuchi, Tanaka	1	12	N/a	Growth Hormone	Concave facial profile, bulging forehead, retrognathic maxilla. Underdevelopment of the nasomaxillary complex. Anterior crossbite; molar relationship was bilaterally Angle Class III. Open bite. Maxillary dental midline was deviated 6.5 mm to the left toward the facial midline	Maxillary protraction with headgear + orthodontic sectional arch	y	y	n	n	83 months (multibracketing)	ANB, −8.0 SNA, 62.5 (S‐N) was more than 5 SD greater than the norm. The mandibular plane angle was within the normal range (FMA, 28.5) The maxillary incisors were labially inclined (U1‐SN, 125.1), and the mandibular incisors had a lingual incli‐nation (L1‐MP, 87.6)	The ANB angle decreased from 8.0 to 6.0, indicating that the skeletal jaw‐base relationship was still Class III. The maxillary incisors still inclined labially (U1‐SN, 120.8), and overjet and overbite were 14.7 and 1.5 mm, respectively (Table). The maxillary dental midline still deviated 4.0 mm to the left compared with the facial midline; however, due to lateral expansion, arch length discrepancies were 12.0 mm for the maxilla and 1.0 mm for the mandible Class I	Six years after retention (age 28 years 5 months), an acceptable occlusion was maintained without any recur‐rences, indicating long‐term stability of the occlusion (Figs 9–11). The cephalometric analysis showed considerable forward‐downward mandibular growth, resulting in a 1.2 increase of the SNB angle and a 1.2 decrease of the ANB angle		Orthopaedic/orthodontic.
**Ohba**, Tenshin, Takano‐Yamamoto	1	10	Female	Growth hormone	Class II division 1; concave profile. 2 mm midline deviation. Skeletal Class III	Orthodontic bracketing. Occipital headgear. 4 first premolars extraction	y	y	4 premolars	n	41 months	Ceph pre post; SNA	Class I with normal overjet and overbite. Concave profile not corrected, unchanged. Midline deviation corrected.	2 years, stable	Poor	Orthodontics/orthopaedics
**Celenk**, Arici	1	16	Male	Lengthening of the lower limbs; Myringotomy and tube placement	Concave facial soft tissue profile, macroglossia, buccal cross‐bite and a posterior open bite. Class III molar relation	Orthopaedic expansion (Hyrax); Bracketing	y	y	no	no	24 months	Ceph but no data	Malocclusion was corrected and the posterior open bite closed	No		Orthodontics
**Karpagam**, Rabin, George, Santhosh	1	14	Female		Anterior open bite 5 mm, labial incompetency, Molar class I, enamel hypoplasia; 22 agenesis	Orthodontic presurgical and surgical vertical osteotomy	No	y	Yes 2 lower premolars, and 1 upper	y		SNA 73°, distance of NA perpendicular to A of 9 mm (mean 0‐lmm.) and FH‐NA value of 80° (mean 88°). Mandible and chin position were normal with SNB of 78° and facial angle of 88′ (mean 88°). AB—NPog measuring 5′ (mean 0′) and ANB of—5° indicated the presence of skeletal class III malocclusion. Facial axis angle of 84° and FMA 37 revealed a vertical growth	Open bite correction, normal overjet and class I. Midline correction	LeFort I maxillary superior impaction (followed by autorotation of mandible) was planned to be done in two years time, after cessation of any possible growth of the maxilla	Hyperactive	Orthodontics; maxillofacial surgery
**Dunbar**, Goldin, Subtelny	1	11	Female	Tonsillectomy and myringotomy	Posterior cross bite, class I	Hyrax, facemask, brackets, lip bumper.	y	y	Yes 1 lower lateral incisors	no	42 months	Skeletally the chin was slightly forward with a facial angle of 87″ when compared with the Michigan mean of 84.6″ at age 11. The maxilla was positioned posteriorly to nasion with a 79″ FH‐NA angle (mean 88″). The profile was concave with a—12″ angle of convexity. Radiographic evaluation of the adenoids showed approximately 75% airway obstruction. The frontal radiograph showed slight maxillary constriction. Nasal width was within normal limits, bnt cloudiness in the airway was apparent. The denture bases basically mirrored their skeleta; bases with an SNB of 81″ (mean 77.3″), an SNA of 74″ (mean 81.l″), and an ANB of 7″ (mean 3.8″). These findings, along with an A‐B/NPg of 6″ (mean −5.9″): indicated a poor. The mandibular plane relative to the mean, 26″ versus a mean of 28.8″. Dentally the maxillary incisors were prochned relative to the SN plane and Frankfort horizontal plane? 16.5″ and 122″, respectively, with means of 105.1″ and 112.5″. Relative to the palatal plane, the upper incisor was well related due to the upward anterior palatal tilt. The mandibular incisors were slightly retroclined relative to the mandibular plane at 85.5″ (mean 93.3″). The incisal edges of both maxillary and mandibular incisors were posi‐ tioned anteriorly relative to the A‐PO plane at 8 mm and 5.5 mm, respectively, with means of 6.5 mm and 1.6 mm	A very acceptable occlusion. A super Class I molar relationship with a Class III tendency at the canines has resulted. This is primarily attributable to a deficient maxilla. All teeth are in the arch and in good alignment (Fig. 5). Cephalometric analysis at retention. At age 15, the chin to forehead relationship has remained relatively stable and now is slightly posterior to the Downs mean, 86″ versus a mean of 87.2″. The anteroposterior position of the maxilla has decreased 2″ to 77″ for the FH‐NA angle; the angle of facial convexity has become slightly more concave. The man‐dibular plane has remained reasonably stable with an increase of 1.5″. The vertical dimension has remained relatively constant, although the maxilla appears to have moved slightly inferiorly relative to the anterior tubercle of the atlas. The upper facial height to total facial height ratio is virtually unchanged. Dentally the proclination of the maxillary incisors is un‐ changed, while the mandibular incisors are slightly proclined, from 85.5″ to 88″ to the mandibular plane. The maxillary incisors have increased from 8 mm to 13 mm relative to the APo plane (mean 5.7 mm). The interincisal angle has become more acute, decreasing from 127″ to 122″ (mean 131.9″) (Fig. 6 and Table I)	3 years, stable		Orthodontics
**Elwood**, Burstein, Graham, Williams, Paschal	2	3 yo; 6 yo.	Female; Male	Tracheostomy	Midface hypoplasia	Midfacial distraction. Modified Lefort I osteotomy with placement of bi‐ lateral midface internal expanders. 25 mm midface distraction	n	n	n	y	42		A class II occlusal relationship without open bite was created. He remains asymptomatic after 2 years of follow‐up. The initial overjet of the maxillary incisors has gradually decreased with mandibular growth	18 months—2 years		Maxillofacial/Orthodontic
Denny, A. D., Gingrass, D. J., & Ferguson, D. J.	1	15	N/a	Previously managed inadequately by nonextraction orthodontics.	Severe Angle's class III malocclusion; 5‐mm overjet	Lefort I surgery, tip rhinoplasty and replacement of the frontal bone loss using cranial bone graft was performed.	n	n	maxillary first permanent premolars	y	36 months	The maxillary incisors were tilted excessively to an unstable 145 degrees relative to line anterior nasal spine‐posterior nasal spine (ANS‐PNS). The occlusal plane was tipped inferiorly from ANS to PNS and was measured at 1–8 degrees below the SN‐7 reference line	Good aesthetics and facial results	No		Maxillofacial/Orthodontic
Govindraj, V., Datana, S., Sharma, M., & Chauhan, D.	1	23	Female	Extractions	Incompetent lips (short upper lip with increased interlabial gap of 18 mm), complete incisal show with 6 mm of gingival visibility, centered maxillary dental midline, and mandibular midline shifted to the right by 2 mm. Multiple missing teeth with a history of extraction due to caries, multiple restorations, constricted maxillary arch, palatally erupted 12 and 22 with increased overjet of 12 mm, overbite of 25%, nonspecific molar, and canine relation bilaterally	Pre surgical stage 24 months; Le Fort I (LF‐1) osteotomy with 6 mm maxillary superior repositioning followed by 5 mm bilateral sagittal split ramus osteotomy (BSSRO) with anticlock wise rotation of mandible to achieve optimal overjet and overbite. Stage III: Settling of occlusion and fixed retention in maxillary and mandibular arch followed by rehabilitation of missing 35	n	y	n	y	30 months	Cephalometric analysis of lateral cephalograph confirms hypoplastic maxilla, orthognathic mandible, skeletal Class III bases, maxillary dentoalveolar protrusion with supraerupted incisors, and upright mandibular incisors with supraerupted mandibular incisors and molars	Improved facial aesthetics with harmonised smile, boosting the patients morale and confidence as conveyed by patient herself	6 months	Normal	Maxillofacial/Orthodontic
Lueveswanij, S., & Nuntanaranont, T.	1	18	Female	Previous orthodontic treatment for approximately 30 months. Medical history was unremarkable. Growth hormone therapy was refused.	Anterior open bite. Maxilla was hypoplastic Midface deficiency Mandible was relatively prognathic with an anter‐ ior open bite and protrusion of the lower lip Incompetent lips and a tongue‐ thrust swallowing pattern Class III malocclusion with an anterior open bite was noted	Mandibular surgery, orthodontics, extractions	n	y	Bilateral lower premolars	y		Measurement SNA 82 Co‐A 86 SNB 84 ANB‐2 SNMP 39	One year after surgery, a more acceptable facial appearance and normal function of the stable dental occlusion were noted (Figures 2b and 3). Accordingly, her phonetic problem had reportedly resolved	1 year	Sociable	Maxillofacial/Orthodontic
Kau, C. H., Vincent, J., Oberoi, S., Kau, S. Y. C., & Waite, P. D.	1	19	Male		Anterior crossbite with −9 mm overjet, left side posterior crossbite, and significant deep overbite. Midline was coincident. There was no popping, clicking, or crepitation of the temporomandibular joints. Molar and canine class III with anterior and posterior left transverse discrepancy	Surgery First (ortho+ surgery) non‐extraction treatment, followed by a Le Fort 1 maxillary osteotomy to advance the maxilla, bilateral sagittal split setback with intermaxillary fixation screws. Post‐surgical orthodontic treatment with the use of conventional orthodontic brackets was carried out to correct inter‐arch discrepancies after the surgery	n	y	n	y	7 Months	Pre‐treatment Post‐treatment Normal Skeletal Sella‐nasion‐A point (°) 73.9 81.7 82.0 Sella‐nasion‐B point (°) 89.0 88.3 80.9 Point A‐nasion‐B point (°) −15.1 − 6.6 1.6 Skeletal vertical Mandibular plane—sella‐nasion (°) 25.1 26.6 33.0 Frankfort horizontal plane—mandibular plane angle (°) 26.4 29.2 23.9 Dental Overjet (mm) −9.0 2.0 2.5 Overbite (mm) 8.0 1.8 2.5 Upper incisors—sella nasion (°) 124.7122.8102.8 Upper incisors—nasion‐A point A (mm) −16.8 15.0 4.3 Lower incisors—nasion‐point B (mm) 4.6 4.4 4.0 Lower incisors—mandibular plane (°) 85.4 86.0 95.0 Soft tissue Lower lip to E‐plane (mm) 4.5 5.6–2.0 Upper lip to E‐plane (mm) −1.6 2.7–6.0	Class I molar and canine relationship, normal overbite and overjet were achieved with coincident dental centerlines to the mid‐sagittal plane	No		Maxillofacial/Orthodontic
Gurdán, Z., Szalma, J., & Benedek, P.	1	11	Female	Ventilation tube, ureteral stone, tympanostomy tubes, orthopaedic treatment hinged plastic splints for the knees, Ilizarov devices limbs, CPAP therapy	Angle classification, (‘class II/2’) discrepancy, there was also a vertical (minor open bite) and a transverse (deviation of the upper midline to the right by 4 mm) dental anomaly, transversal constriction	Fixed screw rapid expander upper appliance, Hyrax; fixed appliance	y	y	Extract the upper first premolars	n	24 + months		Harmonising the dental arch and establishing occlusion	No	Good	Orthopaedic/orthodontic.
Paul, Rosaline Tinaa; Biswas P.P.b; Nandan, Hemantd; Ligil A.R.a; Thanikunnel, Joseph K.c	1	14	Female		Class III		n	y	First premolars	n		Parameters 2. SNB 4. Mandibular plane Dental parameters 2. UI to NA (linear) 4. LI to NB (linear) Soft tissue parameters 2. Lower lip to S line months follow up lateral cephalogram and OPG Pretreatment 1. SNA 82 0+/−2 78.2 0 78.8 0 Retrognathic maxilla 80 0+/−2 32 0+/−4 4 mm 4 +/−2 mm 78.5 0 41.7 0 16.5 mm 8.7 mm 6.8 mm Posttreatment 78 0 42.6 0 13.7 mm 8 mm 5.2 mm Orthognathic mandible Dolichocephalic Protrusion Protrusion Protrusive lips 3. ANB 3 0+/−2 −1.3 0 0.8 0 ClassIIIskeletalpattern 5. Occlusal plane 14 0+/−4 25.4 0 29.4 0 Clockwise rotation 1. UI to NA (angle) 22 0+/−2 47.8 0 33.1 0 Vestibular version 3. LI to NB (angle) 25 0+/−2 46.2 0 26.7 0 Vestibular version become normal 5. Interincisal angle 1310+/−6 87.2 0119.3 0 Protrusion 1. Upper lip to S line 0 mm 6.0 mm 5.2 mm Protrusive lips	The end results were satisfactory both extraorally and intraorally and the patient appeared to pose a confident smile	7 months		

A PRISMA flow diagram summarising the selection process is presented in Figure 1.

### 3.2 Overview of Included Studies

Among the 13 included papers the majority (*n* = 11) were single‐case reports [11, 19, 22, 23, 24, 25, 26, 27, 28, 29, 30, 31, 32, 33]. A smaller subset (*n* = 2) reported on multiple patients (≥ 2 patients, but with no controls) presenting case series design [18, 19, 20, 21, 22, 23, 24, 25, 26, 27, 28, 29, 30, 31, 32, 33, 34].

Other Publication Types (e.g., short letters, textbook chapters, editorials): These were generally excluded or had limited data to be included in the final selection, and especially did not include a detailed description of the treatment, nor the follow‐up.

The publications found were originally published from 1989 to 2023, with most articles published in the past two decades. Geographically, studies originated from various regions (North America, Europe, Asia, the Middle East), showing a broad international interest in achondroplasia management.

### 3.3 Participants Demographics and Age

All included studies involved patients carrying a confirmed genetic diagnosis of achondroplasia (ACH). Age Range at Treatment: Paediatric cases typically ranged from 6 to 14 years, a cohort of younger patients was also included ranging from the age of 3 years to 6 years, whereas some reports also addressed adult patients (18+) a threshold of 16 years can be used to distinguish predominantly growth‐modifiable from post‐growth treatment phases. A few documented older adolescents or young adults up to 23 years. Limited details were provided, but overall there was a partially equal male–female distribution across the combined dataset; several papers (especially older case reports) did not explicitly mention sex of the patients.

### 3.4 Study Designs and Treatment Types

All included studies were observational (case report/series). No randomised controlled trials (RCTs) or large cohort studies were identified, reflecting the rarity of clinically oriented ACH studies and the typical reliance on individual or small‐group reports.

#### 3.4.1 Orthopaedic and Orthodontic Interventions

Rapid Maxillary Expansion (RME) was described in several paediatric cases to address midfacial deficiency and transverse maxillary constriction. RME often coincided with positive airway changes (improvements in nasal airflow), although the follow‐up periods were medium to short (6–24 months).

Functional Appliances (facemask or removable functional appliances) were occasionally used in younger patients showing a Class III tendency from midface hypoplasia, showing great results.

#### 3.4.2 Orthodontic Mechanics

Fixed Appliance Therapy: Many case reports showed comprehensive fixed appliances (brackets and wires), but they were always combined with surgical extraction or maxillofacial surgery.

Extraction vs. Non‐extraction: Decisions typically depended on crowding severity and skeletal discrepancy. Several authors noted caution with extractions in ACH, where midfacial deficiency might be exacerbated by further retraction of anterior teeth, while some others adopted a maxillofacial surgical approach, while a few studies included both extraction and maxillofacial surgery.

#### 3.4.3 Surgical Interventions

Orthognathic Surgery: Some adolescent or adult patients underwent maxillary advancement (Le Fort I) or bimaxillary procedures to correct skeletal Class III or severe open bite.

Midface Distraction was reported in a small subset (2‐patient case series) addressing airway compromise and severe midfacial hypoplasia. “Surgery‐First” Approach: One recent case (Kau et al. [31]) applied a surgery‐first protocol for time efficiency, demonstrating short‐term success but lacking long‐term data. Overall, the primary rationale was to correct Class III relationships, manage crowding, or expand the transverse dimension and mitigate airway issues.

### 3.5 Follow‐Up Duration and Outcomes

Short‐Term Follow‐Up (6–12 months) was most common. Several reports described treatment completion but gave limited post‐treatment stability data.

Medium‐Term Follow‐Up (up to ~24–36 months) appeared in a few older case reports (e.g., Dunbar et al. [26], with ~3‐year follow‐up) and in some surgically treated cases.

Long‐Term Data (beyond 3 years) were rarely documented, limiting conclusions about stability.

Reported Outcomes:

Dental: Improved occlusion, alignment, resolution of crowding or crossbite.

Skeletal: Increased SNA angle (maxillary advancement) or improved midfacial contour in surgical/distraction cases.

Airway: Some studies reported subjective improvements in nasal breathing or polysomnography changes (when airway compromise was documented), although objective measures were rarely standardised.

### 3.6 Risk of Bias (R.O.B.)

Using the Joanna Briggs Institute (JBI) Critical Appraisal Tools:

Case Reports (11 Items): Most achieved a “Yes” for describing patient demographics, clinical details, and interventions. However, adverse events were often unclear or not reported, and longer‐term stability was rarely addressed.

Case Series: Most lacked explicit inclusion criteria, had very small samples (often 1–5 patients), and did not address whether cases were consecutive or if any were excluded. Statistical analyses were often minimal or absent. The included studies largely fell under a moderate to high risk of bias. The single‐patient designs inherently limit generalizability, and methodological details (ethics approval, adverse event tracking) were often incomplete or missing.

In general, the tool described a low to medium risk of bias, with some moderate/high exception, the most common missing item was the lack of adverse events description, followed by the lack of follow‐ups and the lack of systemic conditions of the patient. However, in general, the case reports can be described as mostly reliable for this type of study design. All the results were synthesised in Table 2. Since most of the studies had similar scores, all were considered for the final conclusions taken.

**TABLE 2:** Shows the result of the Joanna Briggs Institute (JBI) tool and the detailed results for each selected study.

Citation	Study type	Key JBI indicators (yes/no/unclear/N/A)	Key limitations	Overall RoB
Pineau M, Farrow E, Nicot R, Ferri J. (2018) *Achondroplasia: Orocraniofacial Features…*	CS and narrative	1–6;8: yes 7: no	– The case series is well presented and aligns with most domains. – Main limitation: Lack of explicit mention of adverse or unexpected outcomes.	Low
Mori H, Matsumoto K, Kawai N, Izawa T, Horiuchi S, Tanaka E. (2017) *Long‐term follow‐up…*	CR	1–6;8: yes 7: partially	– Single case. – The longitudinal follow‐up and cephalometric are well documented.	Low/Moderate
Ohba T, Ohba Y, Tenshin S, Takano‐Yamamoto T. (1998) *Orthodontic treatment of Class II…*	CR	1–6;8: yes 7: no	– Single case report; it fulfils most criteria providing non‐surgical management of a Class II profile in an achondroplasia patient, highlighting the role of early functional therapy.	Low/Moderate
Celenk P, Arici S, Celenk C. (2003) *Oral Findings in a Typical Case…*	CR	1–6;8: yes 7: no	– Single patient. – 24‐month active treatment, unclear long‐term stability beyond that. – No explicit statement of consent/ethics.	Moderate
Karpagam S, Rabin K, George M, Karthikeyan K. (2005) *Correction of anterior open bite…*	CR	1–4;8: yes 5–6: partially 7: no	– A well‐structured case report addressing a complex dentofacial presentation in achondroplasia with innovative staged management. – Strong in surgical detail, treatment rationale, and follow‐up interpretation. – Explicit adverse event reporting is lacking, the discussion acknowledges clinical limitations and the need for further intervention, which partially satisfies this domain.	Moderate/High
Dunbar JP, Goldin B, Subtelny JD. (1989) *Correction of Class I crowding…*	CR	1–6;8: yes 7: no	– Case report with longitudinal follow‐up, comprehensive cephalometric data, and strategic non‐surgical approach. – The lack of adverse effects is the only modest limitation.	Low/Moderate
Elwood ET, Burstein FD, Graham L, et al. (2003) *Midface Distraction to Alleviate Upper Airway…*	CS (2 patients)	1–6;8: yes 7: no	– 2 patients, no control group. – Follow‐up ~18–24 months.	Low
Denny AD, Gingrass DJ, Ferguson DJ. (1992) *Comprehensive correction…*	CR	1–5;8: yes 6: partially 7: no	– Single patient; no long‐term follow‐up beyond immediate post‐op. – No explicit mention of informed consent.	Moderate
Govindraj V, Datana S, Sharma M, Chauhan D. (2022) *Craniofacial Manifestation and Orthosurgical…*	CR	1;3–6;8: yes 2: partially 7: no	– Single case, short follow‐up (6 months). – Generalizability limited; no mention of IRB/consent specifics.	Moderate
Lueveswanij S, Nuntanaranont T. (2002) *Orthognathic Surgery in Achondroplasia…*	CR	1–6;8: yes 7: partially	– Single patient, only 1‐year follow‐up. – Lack of detailed adverse event reporting	Moderate/Low
Kau CH, Vincent J, Oberoi S, Kau SYC, Waite PD. (2023) *Surgery first approach…*	CR	1–5;8: yes 6;7: partially	– Case report featuring modern technologies (VSP, 3D CBCT, CAD/CAM), interdisciplinary planning, and a patient‐centered approach. – Minor limitation in the lack of discussion of complications. – Long‐term stability with SFA unknown.	Moderate
Gurdán Zs, Szalma J, Benedek P. (2021) *Az achondroplasia a fogszabályozás…*[Hungarian]	CR	1–5;8: yes 6;7: partially	– Single case; no extended posttreatment data. – Focus on airway improvement more than final occlusal stability. – Full text in foreign language (Hungarian)	Moderate/High
Rosaline Tina Paul, Biswas PP, Ligil AR, et al. (2021) *Achondroplasia—Differential Diagnosis…*	CR	1–6;8: yes 7: partially	– Single patient, 7‐month follow‐up only. – Integration of orthodontic, genetic, and systemic findings. – No explicit mention of ethics approval.	Low/Moderate

### 3.7 Preliminary Guidelines

Functional orthopaedic treatment plus orthodontics was successful in most III class cases: 12 yo described by Mori et al. 11 yo by Dunbar et al., 11 yo Gurdàn et al. up to the age of 16 treated by Celenk et al.; also it was described as successful in one II class, 10 yo by Ohba et al. It resulted unsuccessful in one male who was treatment with maxillary fixed plates (11 y.o) by Pineau et al. in a case series composed of three patients in total. These patients benefited from early orthopaedic protraction with facemask or expansion appliances, followed by multibracket systems or fixed appliances. Out of those patients 3 of them were followed for long term stability with good results.

The patients who did not receive functional treatment were all older than 12, out of which all of them (8 in total) reported good results, and malocclusion resolution but only two of them reported follow‐ups. This group, however, had to be treated surgically with extractions in 4 cases, and maxillofacial surgery in 5 cases, no case sustained orthodontics without surgery nor extractions, but one case sustained only 1 tooth extraction (lower later incisor). What is not known to the researchers and cannot be drawn from the studies selected is if surgery was planned as part of the treatment, or if orthodontics failed and surgery was required after.

Finally, the latest group which sustained maxillofacial only treatment achieved good results: early treatment of airway obstruction using midface surgical distraction in very young children (Elwood et al. ages 3 and 6) resulted in decannulation of tracheostomies and marked improvement in upper airway dimensions, underscoring the functional priority of surgical intervention in obstructive sleep apnea (OSA) cases. A summary table resumes the collected evidence (Table 3).

**TABLE 3:** Shows the summarised evidence of the treatment possibilities during the different stages of growth.

Group	Age range	No. of cases	Representative studies	Treatment	Outcome	Maxillofacial surgery or extraction required
Functional Orthopaedics + Orthodontics	10–16 years	5	Mori, Dunbar, Gurdàn, Celenk, Ohba	Facemask OR RME + multibracket systems	Successful; 3 had long‐term stable results	No
Orthodontics + Maxillofacial Surgery OR Extractions	> 12 years	8	Karpagam, Kau, Govindraj, Rosaline, Denny, Lueves	Orthodontics + extractions and/or surgery	Good short‐term outcomes; only 2 long‐term follow‐ups	Yes – 4 extractions, 5 surgeries
Surgery Only (Airway Focused)	3–6 years	2	Elwood	Midface distraction	Successful decannulation; airway improved	Yes – distraction osteogenesis

The following guidelines can be drawn: early orthopaedic intervention using facemask or rapid maxillary expansion combined with multibracket orthodontics demonstrated consistent success in patients aged 10–16 years with Class III, Class II, and even Class I malocclusions. Among these cases, most achieved favourable outcomes, with long‐term stability confirmed in several patients over 16 years of age required more invasive approaches, either extractions or maxillofacial surgery, to achieve malocclusion correction. Although short‐term outcomes in this older cohort were satisfactory, long‐term follow‐up was reported in only a minority of the studies.

Several studies reported improvements in airway‐related symptoms following orthopaedic or surgical interventions, particularly rapid maxillary expansion and midface distraction. However, airway outcomes were predominantly described qualitatively. Objective measurements such as polysomnography, rhinomanometry, or standardised imaging‐based airway volumetry were inconsistently reported and not comparable across studies [35]. Overall, these findings were synthesised in a age‐stratified treatment planning: non‐surgical orthopaedic–orthodontic therapy is generally described as effective and stable in younger individuals, while combined surgical approaches appears to become necessary in post‐growth patients.

## 4 Discussion

The present systematic review provides the most up‐to‐date synthesis of orthopaedic, orthodontic and orthognathic interventions in achondroplastic individuals, encompassing 13 publications (11 single‐case reports and 2 small case series) that span more than three decades. Although the heterogeneity and small sample sizes did not allow quantitative pooling, several clinically relevant patterns emerged which were collected in a comprehensive guideline. More efforts are needed to increase the validity of the craniofacial treatment on ACH patients and to provide solid guidelines for the clinicians [36, 37, 38, 39]. The guidelines should be treated as starting point extracted from a 3 decades long literature for a rare condition, and not as an absolute mean in decision making, whereas different factors and the variability of syndromes should be taken with care. In parallel, future research should explore the potential synergistic effects of emerging pharmacological therapies, such as vosoritide, when combined with orthodontic or maxillofacial surgical interventions. However, given that vosoritide received FDA approval only recently (2021–2023), it remains premature to assess its impact on orthodontic treatment completion, which typically requires multi‐year follow‐up to reach definitive outcomes. Airway outcomes represent a clinically relevant but under‐quantified domain in the available literature. Most included studies relied on subjective symptom improvement or clinically‐oriented indicators or symptoms analysis.

Given the premises, we could synthetize a few useful recommendations. Interceptive orthopaedic/orthodontic protocols applied during the first and early second decades of life (10–16 years) yielded reproducibly favourable outcomes. Facemask protraction or rapid maxillary expansion (RME) followed by comprehensive fixed appliances corrected Class III, Class II and Class I malocclusions in 5 of 6 analysed patients, with documented long‐term stability in three cases [40, 41, 42]. These findings reinforce the biological advantage of utilising the remaining mid‐facial growth potential before synchondrosis closure and mirror principles long applied to non‐syndromic Class III correction. Evidence from adolescent cohorts indicates that early correction of maxillary constriction can improve occlusal and functional outcomes and should inform timing decisions in syndromic patients [43]. However, it should be treated with caution as some patients were described as hyperactive or presenting low compliance, which is fundamental in this type of treatment [44, 45].

By contrast, patients treated after cessation of active growth (> 16 years) almost invariably required either tooth extractions or maxillofacial surgery (or even both in one case). While short‐term occlusal goals were achieved, only two reports provided follow‐up beyond 24 months, leaving stability uncertain. Mid‐facial hypoplasia and a constricted nasopharyngeal airway are hallmarks of achondroplasia. Two early‐childhood cases (3 y and 6 y) treated with midface distraction demonstrated decannulation and polysomnographic normalisation, underlining that airway patency may supersede occlusal objectives in very young, symptomatic patients, but if they are present in mild manifestations they can be improved with orthopaedic/orthodontic treatment. In fact, in the orthodontic cohort RME frequently produced subjective or rhinometric improvements in nasal airflow, although airway assessment tools and length of observation were inconsistent across studies. Future reports should incorporate standardised polysomnography or cone‐beam CT volumetry to quantify airway volume and to register improvements [46].

From the pharmacological side, only two publications explicitly reported recombinant growth‐hormone administration during dental treatment, and no study included patients receiving the recently approved C‐type natriuretic peptide analogue, vosoritide. Given the accelerating uptake of pharmacological growth modulation, prospective registries are needed to clarify whether altered craniofacial trajectories mandate protocol adjustments or modified retention schemes [13].

Application of the JBI critical appraisal tools revealed a predominantly moderate risk of bias; deficiencies most frequently related to incomplete adverse‐event reporting, short follow‐up periods, and the absence of consecutive patient inclusion. Patient‐reported outcome measures (PROMs) and satisfaction are important endpoints in craniofacial interventions and should be included where available [47]. While case reports and case series remain invaluable in rare disorders, their imitations necessitate very cautious extrapolation. Rare‐disease case reports, in fact, are inherently prone to positive‐outcome bias, and should be taken with care. Furthermore, the nature of the studies limits the presence of standardised outcome measures (such as cephalometric endpoints, airway indices, or patient‐reported outcomes) over more than three decades. For what concerns the respiratory results standardised outcomes such as polysomnography (AHI, oxygen desaturation index), CBCT‐based upper airway volumetric analysis should be included in future prospective studies and registries.

Multicentre prospective cohorts with uniform outcome metrics and longer follow‐ups would upgrade the overall evidence.

### 4.1 Implications for Practice and Guidelines

Synthesised evidence supports a staged approach (Table 4):

**TABLE 4:** shows the collected evidence and guidelines for the treatment of the Achondroplastic patient.

Age range	Treatment	Class	Expected outcome	Maxillofacial surgery or extraction required
10–16 years	Facemask OR RME + multibracket systems	III	Successful 3/4; 2/4 had long‐term stable results	No
10–16 years	Facemask OR RME + multibracket systems	II	Successful 1/1 with long‐term stable results	No
10–16 years	Facemask OR RME + multibracket systems	I	Successful 1/1 with long‐term stable results	No
> 16 years	Orthodontics + extractions and/or surgery	III	Good short‐term outcomes; 2 long‐term follow‐ups confirmed	Yes, extraction 4/9 or maxillofacial surgery 5/9
> 16 years	Orthodontics + extractions and/or surgery	I	Good short‐term outcomes 1/1.	Yes, maxillofacial surgery 1/1
< 6 years	Midface distraction	N/a	Successful decannulation; airway improvement	N/a

≤ 16 years: early RME ± facemask to harness growth, minimise need for extractions, and potentially mitigate obstructive sleep‐apnoea risk.

> 16 years: combined orthodontic–surgical plans should be anticipated and discussed early with patients and families, emphasising realistic expectations regarding stability and airway benefits.

Any age with severe airway compromise: priority consideration for maxillofacial advancement or distraction, even in the primary dentition, within a multidisciplinary framework including ENT and sleep‐medicine specialists.

## 5 Conclusions

Growth‐guided orthopaedic protocols in achondroplastic paediatric and adolescent patients reliably correct transverse and sagittal discrepancies with less need for surgery. Early maxillary protraction should be considered before cranial‐base synchondrosis closure to exploit residual growth potential. After growth completion, orthodontic decompensation alone is seldom sufficient; Le Fort I advancement, bimaxillary procedures, or distraction osteogenesis become integral to comprehensive care along with possible extractions. Virtual surgical simulation is recommended to anticipate segment stability in a hypoplastic mid‐face. In cases with documented obstructive sleep apnoea, midface advancement can be considered in early age, routine polysomnography is advised prior to initiating maxillary expansion, given the high prevalence of sleep‐disordered breathing.

However, the current literature is mostly confined to case report evidence designs, with scarce long‐term stability data and no information on patients undergoing modern biological therapies for this reason the underlying evidence is low. Patients receiving pharmacological treatment should undergo frequent cephalometric or tridimensional CBCT monitoring until growth completion. High‐quality prospective trials and registries are needed.

## Author Contributions

Conceptualization: M.F. Data curation: M.F. Formal analysis: M.F. Funding acquisition: G.M.T. Investigation: M.F.; C.M. Methodology: M.F. Project administration: M.F.B.; B.R. Resources: G.M.T.; B.R. Software: C.G. Supervision: M.F.B. Validation: M.F.B.; B.R. Visualisation: C.G. Writing – original draft: M.F. Writing – review and editing: G.M.T.; C.M.

## Funding

The authors have nothing to report.

## Ethics Statement

Not required for this study as it is a systematic review based exclusively on previously published studies and does not involve human participants or the use of identifiable personal data.

## Conflicts of Interest

The authors declare no conflicts of interest.

## Supporting information


**Data S1:** ocr70108‐sup‐0001‐supinfo.docx.

## Data Availability

The data that support the findings of this study are available from the corresponding author upon reasonable request.
