# Supplementary material for: Orthodontic and Maxillofacial Surgery Treatment in Achondroplasia for Orofacial Alterations: A Systematic Review and Preliminary Age‐Stratified Guidelines
Source: Orthod Craniofac Res. 2026 Apr 6;29(4):593–610. doi: 10.1111/ocr.70108 (PMC13485178; doi:10.1111/ocr.70108)
Supplement: Supplementary file 1 [file OCR-29-593-s001.docx]

| **Meta analysis** | **Primary study** | **Surgical technique** | **Number of implants** | **Platform** | | **Apex** | |
| --- | --- | --- | --- | --- | --- | --- | --- |
|  |  |  |  | **Mean** | **SD** | **Mean** | **SD** |
| Khaohoen et al. | Smitkarn et al. 2019 [7] | s-CAIS (full) | 30 | 1 | 0.6 | 1.3 | 0.6 |
| 2025 [1] |  | Freehand | 30 | 1.5 | 0.7 | 2.1 | 1 |
|  | Vercruyssen et al. 2014 [8] | s-CAIS (full) | 52 | 1.38 | 0.64 | 1.6 | 0.7 |
|  |  | s-CAIS (full) | 49 | 1.33 | 0.82 | 1.5 | 0.72 |
|  |  | s-CAIS (part) | 55 | 1.23 | 0.6 | 1.57 | 0.71 |
|  |  | s-CAIS (part) | 53 | 1.6 | 0.92 | 1.65 | 0.82 |
|  |  | Freehand | 51 | 2.77 | 1.54 | 2.91 | 1.52 |
|  |  | s-CAIS (part) | 51 | 2.97 | 1.41 | 3.4 | 1.68 |
|  | Varga et al. 2020 [9] | Freehand | 18 | 1.76 | 0.7 | 2.41 | 0.9 |
|  |  | s-CAIS (part) | 20 | 1.38 | 0.63 | 1.72 | 0.77 |
|  |  | s-CAIS (part) | 17 | 1.51 | 0.73 | 1.83 | 0.71 |
|  |  | s-CAIS (full) | 15 | 1.54 | 0.39 | 1.53 | 0.54 |
|  |  | Freehand | 37 | 1.84 | 1.05 | 2.3 | 1.02 |
|  |  | s-CAIS (part) | 29 | 1.69 | 1.06 | 1.95 | 1.28 |
|  |  | s-CAIS (part) | 34 | 1.21 | 0.73 | 1.41 | 0.83 |
|  |  | s-CAIS (full) | 37 | 1.34 | 0.58 | 1.62 | 0.61 |
|  | Lou et al. 2021 [10] | Freehand | 30 | 1.05 | 0.17 | 1.36 | 0.13 |
|  |  | s-CAIS (full) | 33 | 0.39 | 0.12 | 0.28 | 0.09 |
|  |  | s-CAIS (part) | 36 | 0.69 | 0.1 | 0.8 | 0.08 |
|  | Wei et al. 2022 [11] | d-CAIS | 12 | 1.01 | 0.41 | 0.88 | 0.43 |
|  |  | Freehand | 12 | 1.51 | 0.67 | 1.94 | 0.86 |
|  | Yotpibulwong et al. 2023 | Freehand | 30 | 1.48 | 0.68 | 2.18 | 0.95 |
|  | [12] | s-CAIS (full) | 30 | 1.06 | 0.67 | 1.4 | 0.71 |
|  |  | d-CAIS | 30 | 1.02 | 0.45 | 1.28 | 0.5 |
|  | Shi et al. 2024 [13] | Freehand | 10 | 1.88 | 1.12 | 2.89 | 1.64 |
|  |  | r-CAIS | 10 | 1.27 | 0.46 | 1.47 | 0.62 |
|  | Yang et al. 2024 [14] | Freehand | 70 | 1.48 | 0.93 | 2.14 | 1.25 |
|  |  | r-CAIS | 70 | 0.76 | 0.36 | 0.85 | 0.48 |
|  | Shen et al. 2015 [15] | s-CAIS (full) | 57 | 1.18 | 0.72 | 1.43 | 0.74 |
|  |  | Freehand | 52 | 2.07 | 0.51 | 2.89 | 1.02 |
|  | Jaemsuwan et al. 2023 [16] | Freehand | 20 | 3.48 | 2 | 3.6 | 2.11 |
|  |  | s-CAIS (full) | 20 | 1.4 | 0.72 | 1.66 | 0.61 |
|  |  | d-CAIS | 20 | 1.73 | 0.43 | 1.86 | 0.82 |
|  | Lorwicheanrung et al. | Freehand | 22 | 3.69 | 1.52 | 3.48 | 1.25 |
|  | 2024 [17] | s-CAIS (full) | 22 | 0.72 | 1.05 | 1.01 | 0.55 |
|  |  | d-CAIS | 22 | 1.12 | 0.43 | 0.98 | 0.37 |
|  | Younis et al. 2024 [18] | s-CAIS (full) | 30 | 0.92 | 0.36 | 1.06 | 0.47 |
|  |  | d-CAIS | 34 | 0.99 | 0.52 | 1.14 | 0.56 |
|  |  | Freehand | 30 | 1.36 | 0.62 | 1.73 | 0.66 |
|  | Alevizakos et al. 2019 [19] | Freehand | 12 | 2.12 | 1.28 | 2.92 | 1.91 |
|  |  | s-CAIS (part) | 8 | 1.26 | 0.68 | 1.47 | 0.67 |
|  | Geng et al. 2024 [20] | Freehand | 30 | 1.28 | 0.12 | 2.22 | 0.3 |
|  |  | d-CAIS | 28 | 0.55 | 0.08 | 0.52 | 0.13 |
|  |  | s-CAIS (full) | 26 | 0.73 | 0.1 | 1.33 | 0.42 |
|  | Li et al. 2024 [21] | Freehand | 40 | 1.29 | 0.52 | 1.78 | 0.59 |
|  |  | s-CAIS (part) | 33 | 1.01 | 0.41 | 1.24 | 0.52 |
|  |  | r-CAIS | 33 | 0.62 | 0.28 | 0.65 | 0.27 |
| Banerjee et al. 2025 [2] | Stafanelli et al. 2020 [22] | d-CAIS | 74 | 0.66 | 0.35 | 1.01 | 0.23 |
| Yin et al. 2025 | Dong Wu et al. 2020 [23] | d-CAIS | 38 | 1.36 | 0.65 | 1.48 | 0.65 |
| [3] | Quan Liu et al. 2022 [24] | s-CAIS (full) | 18 | 0.92 | 0.46 | 1.31 | 0.43 |
|  |  | d-CAIS | 20 | 1.07 | 0.57 | 1.26 | 0.53 |
| Werny et al. | Feng Y et al. 2022 [25] | d-CAIS | 20 | 1.06 | 0.55 | 1.18 | 0.53 |
| 2025 [4] |  | s-CAIS (full) | 20 | 0.99 | 0.63 | 1.5 | 0.75 |
|  | Jorba-García et al. 2023 [26] | d-CAIS | 22 | 1.12 | 0.38 | 1.42 | 0.52 |
|  |  | Freehand | 22 | 1.7 | 0.69 | 2.49 | 1.43 |
|  | Yimarj et al. 2020 [27] | s-CAIS (full) | 30 | 1.04 | 0.67 | 1.54 | 0.79 |
|  |  | d-CAIS | 30 | 1.24 | 0.39 | 1.58 | 0.56 |
|  | Kaewsiri et al. 2019 [28] | s-CAIS (full) | 30 | 0.97 | 0.44 | 1.28 | 0.46 |
|  |  | d-CAIS | 30 | 1.05 | 0.44 | 1.29 | 0.5 |
|  | Magrin et al. 2019 [29] | s-CAIS (part) | 12 | 2.34 | 1.01 | 2.53 | 1.11 |
|  |  | Freehand | 12 | 1.93 | 0.95 | 2.19 | 1 |
|  | Schneider et al. 2019 [30] | s-CAIS (full) | 11 | 0.61 | 0.27 | 1.02 | 0.64 |
|  |  | s-CAIS (full) | 11 | 0.54 | 0.33 | 0.9 | 0.43 |
|  |  | Freehand | 16 | 1.25 | 0.62 | 2.32 | 1.24 |
|  | Younes et al 2018 [31] | s-CAIS (full) | 21 | 0.73 | 0.46 | 0.97 | 0.87 |
|  |  | s-CAIS (part) | 24 | 1.12 | 0.49 | 1.43 | 0.88 |
|  |  | Freehand | 26 | 1.45 | 0.51 | 2.11 | 0.92 |
|  | Block et al. 2017 [32] | d-CAIS | 219 | 1.16 | 0.59 | 1.29 | 0.65 |
|  |  | Freehand | 122 | 1.78 | 0.77 | 2.27 | 1.02 |
|  | Geng et al. 2015 [33] | s-CAIS (part) | 29 | 0.54 | 0.5 | 0.81 | 0.64 |
|  |  | s-CAIS (full) | 30 | 0.89 | 0.78 | 1.1 | 0.85 |
|  |  | s-CAIS (full) | 52 | 0.27 | 0.24 | 0.37 | 0.35 |
|  | Cassetta et al. 2014 [34] | s-CAIS (full) | 225 | 1.68 | 0.6 | 2.19 | 0.83 |
|  | Cassetta et al. 2011 [35] | s-CAIS (full) | 111 | 1.52 | 0.61 | 1.97 | 0.34 |
|  | Cristache et al. 2021 [36] | s-CAIS (full) | 56 | 0.44 | 0.28 | 1.03 | 0.48 |
|  |  | s-CAIS (full) | 55 | 0.85 | 0.44 | 1.48 | 0.72 |
|  | Derkens W et al. 2019 [37] | s-CAIS (full) | 66 | 0.72 | 0.37 | 1 | 0.42 |
|  |  | s-CAIS (full) | 79 | 0.77 | 0.31 | 1.12 | 0.44 |
|  | D'haese J et al. 2012 [38] | s-CAIS (full) | 77 | 0.91 | 0.44 | 1.13 | 0.52 |
|  | Di Giacomo et al. 2012 [39] | s-CAIS (full) | 60 | 1.35 | 0.65 | 1.79 | 1.01 |
|  | Ersoy et al. 2008 [40] | s-CAIS (part) | 94 | 1.22 | 0.85 | 1.51 | 1 |
|  | Fürhauser R et al. 2015 [41] | s-CAIS (part) | 27 | 0.84 | 0.44 | 1.16 | 0.69 |
|  | Gelpi F et al. 2023 [42] | s-CAIS (part) | 40 | 1.08 | 0.59 | 1.77 | 0.81 |
|  | Hanozin B et al. 2022 [43] | s-CAIS (part) | 9 | 1.27 | 0.83 | 2.42 | 1.02 |
|  |  | s-CAIS (part) | 9 | 0.65 | 0.37 | 1.36 | 0.54 |
|  | Kiatkroekkrai et al. 2020 [44] | s-CAIS (full) | 30 | 1.01 | 0.56 | 1.38 | 0.68 |
|  |  | s-CAIS (full) | 30 | 0.87 | 0.49 | 1.1 | 0.53 |
|  | Kraft B et al. 2020 [45] | s-CAIS (part) | 12 | 1.34 | 0.99 | 1.97 | 1.04 |
|  |  | s-CAIS (full) | 12 | 1.26 | 0.57 | 2.5 | 1.67 |
|  | Lee et al. 2013 [46] | s-CAIS (full) | 102 | 1.09 | 1.1 | 1.56 | 1.48 |
|  | Ngamprasertkit C et al. | s-CAIS (part) | 15 | 0.74 | 0.36 | 1.29 | 0.61 |
|  | 2022 [47] | s-CAIS (full) | 15 | 0.48 | 0.22 | 0.71 | 0.31 |
|  | Nickenig et al. 2010 [48] | s-CAIS (part) | 23 | 0.6 | 0.57 | 0.9 | 1.06 |
|  | Orban K et al. 2021 [49] | s-CAIS (part) | 20 | 1.2 | 0.46 | 1.45 | 0.79 |
|  | Ozan O et al. 2009 [50] | s-CAIS (part) | 50 | 1.28 | 0.9 | 1.57 | 0.9 |
|  |  | s-CAIS (part) | 30 | 1.06 | 0.6 | 1.6 | 1 |
|  |  | s-CAIS (part) | 30 | 0.87 | 0.4 | 0.95 | 0.6 |
|  | Pellegrino G et al. 2019 [51] | d-CAIS | 18 | 1.04 | 0.47 | 1.35 | 0.56 |
|  | Schnutenhaus S et al. 2016 [52] | s-CAIS (full) | 12 | 0.9 | 0.5 | 1.5 | 0.7 |
|  | Stübinger S et al. 2014 [53] | s-CAIS (full) | 44 | 0.61 | 0.21 | 0.66 | 0.17 |
|  | Wei et al. 2022 [54] | d-CAIS | 20 | 0.87 | 0.35 | 0.81 | 0.34 |
| Mahardawi et | Huang et al. 2023 [55] | s-CAIS (part) | 24 | 0.48 | 0.26 | 0.97 | 0.39 |
| al. 2025 [5] |  | Freehand | 24 | 1.07 | 0.63 | 1.44 | 0.44 |
|  | Han et al. 2021 [56] | s-CAIS (part) | 50 | 0.74 | 0.21 | 0.81 | 0.16 |
|  |  | Freehand | 52 | 0.87 | 0.39 | 0.93 | 0.25 |
| Luo et al. 2025 | Zhao N et al. 2024 [57] | r-CAIS | 20 | 0.75 | 0.2 | 0.7 | 0.27 |
| [6] | Yu Wu et al. 2024 [58] | r-CAIS | 86 | 0.61 | 0.2 | 0.79 | 0.32 |
|  | Wang M et al. 2024 [59] | r-CAIS | 20 | 0.65 | 0.32 | 0.66 | 0.34 |
|  | Xie R et al. 2024 [60] | r-CAIS | 102 | 0.53 | 0.19 | 0.58 | 0.17 |
|  | Jay M.Neugarten et al. 2024 [61] | r-CAIS | 273 | 1.1 | 0.69 | 1.12 | 0.69 |
|  | Sihui Zhang et al. 2023 [62] | r-CAIS | 62 | 0.68 | 0.36 | 0.69 | 0.36 |
|  |  | d-CAIS | 62 | 1.25 | 0.54 | 1.39 | 0.52 |
|  | Li P et al. 2023 [63] | r-CAIS | 59 | 0.67 | 0.37 | 0.69 | 0.37 |
|  | Wang W et al. 2023 [64] | r-CAIS | 36 | 0.65 | 0.25 | 0.65 | 0.22 |
|  |  | s-CAIS (full) | 48 | 1.37 | 0.72 | 1.28 | 0.68 |
|  | Qiao SC et al. 2023 [65] | r-CAIS | 19 | 0.54 | 0.17 | 0.54 | 0.11 |
|  |  | r-CAIS | 9 | 0.53 | 0.17 | 0.58 | 0.17 |
|  | Chen W et al. 2023 [66] | r-CAIS | 31 | 0.53 | 0.23 | 0.53 | 0.24 |
|  | Jia S et al. 2023 [67] | r-CAIS | 30 | 0.43 | 0.18 | 0.56 | 0.18 |
|  |  | s-CAIS (full) | 30 | 1.31 | 0.62 | 1.47 | 0.65 |
|  | Yang et al. 2023 [68] | r-CAIS | 10 | 0.74 | 0.29 | 0.73 | 0.28 |
|  | Bolding SL et al. 2021 [69] | r-CAIS | 38 | 1.04 | 0.7 | 0.95 | 0.73 |

*Supplementary Table S1 : Dataset of implant deviation measurements (mm) from selected meta-analyses and primary studies, depending on the surgical technique (Freehand ; s-CAIS: static computer-assisted implant surgery (full or partially guided) ; d-CAIS: dynamic computer-assisted implant surgery; r-CAIS: robotic computer-assisted implant surgery).*

| Technique | Parameter | 10,000 runs | 20,000 runs | 50,000 runs | Max variation |
| --- | --- | --- | --- | --- | --- |
| Robotic | Platform | 0.0335 | 0.0334 | 0.0335 | <0.01% |
|  | Apex | 0.0368 | 0.0368 | 0.0368 | <0.01% |
| Dynamic | Platform | 0.0573 | 0.0574 | 0.0573 | <0.01% |
|  | Apex | 0.1102 | 0.1103 | 0.1103 | <0.01% |
| Static full | Platform | 0.1130 | 0.1130 | 0.1130 | <0.01% |
|  | Apex | 0.2360 | 0.2361 | 0.2361 | <0.01% |
| Static partial | Platform | 0.1603 | 0.1604 | 0.1604 | <0.01% |
|  | Apex | 0.2745 | 0.2743 | 0.2744 | <0.01% |
| Freehand | Platform | 0.3177 | 0.3180 | 0.3180 | <0.01% |
|  | Apex | 0.5352 | 0.5350 | 0.5351 | <0.01% |

*Supplementary Table S2 : presents the convergence analysis of Monte Carlo simulations. Probabilities of exceeding the 2-mm safety margin were estimated at 10,000, 20,000, and 50,000 runs for each surgical technique and measurement level. The maximum observed variation across run counts was below 0.01%, confirming the stability of estimates at 20,000 runs.*

References :

[1]	A. Khaohoen, W. Powcharoen, N. Yoda, C. Rungsiyakull, P. Rungsiyakull, Accuracy in dental implant placement: a systematic review and meta-analysis comparing computer-assisted (static, dynamic, robotics) and noncomputer-assisted (freehand, conventional guide) approaches, The Journal of Prosthetic Dentistry 134 (2025) 91.e1-91.e25. https://doi.org/10.1016/j.prosdent.2025.03.038.

[2]	S. Banerjee, A. Debnath, P. Paul, T.N. Banerjee, Comparison of accuracy in freehand versus computer-assisted (dynamic and static) dental implant placement: a systematic review and meta-analysis, J Indian Prosthodont Soc 25 (2025) 22–29. https://doi.org/10.4103/jips.jips_369_24.

[3]	Z. Yin, H. Kuang, L. Li, X. Si, Meta-analysis of the effect of static computer-assisted dental implantation on the accuracy of dental implantation in esthetic area, Afr Health Sci 25 (2025) 343–353. https://doi.org/10.4314/ahs.v25i2.40.

[4]	J.G. Werny, K. Frank, S. Fan, K. Sagheb, B. Al-Nawas, C.T. Narh, E. Schiegnitz, Freehand vs. computer-aided implant surgery: a systematic review and meta-analysis—part 1: accuracy of planned and placed implant position, Int J Implant Dent 11 (2025) 35. https://doi.org/10.1186/s40729-025-00622-w.

[5]	B. Mahardawi, S. Jiaranuchart, S. Arunjaroensuk, K. Dhanesuan, N. Mattheos, A. Pimkhaokham, The accuracy of dental implant placement with different methods of computer-assisted implant surgery: A Network Meta-Analysis of Clinical Studies, Clinical Oral Implants Research 36 (2025) 1–16. https://doi.org/10.1111/clr.14357.

[6]	Z. Luo, A. Li, A. Unkovskiy, J. Li, F. Beuer, Z. Wu, P. Li, Accuracy of robotic computer-assisted implant surgery in clinical studies: a systematic review and meta-analysis, BMC Oral Health 25 (2025) 540. https://doi.org/10.1186/s12903-025-05837-2.

[7] Smitkarn P, Subbalekha K, Mattheos N, Pimkhaokham A. The accuracy of single-tooth implants placed using fully digital-guided surgery and freehand implant surgery. J Clin Periodontol. 2019;46:949–957

[8] Vercruyssen M, Cox C, Coucke W, Naert I, Jacobs R, Quirynen M. A randomized clinical trial comparing guided implant surgery (bone-or mucosa-supported) with mental navigation or the use of a pilot-drill template. J Clin Periodontol. 2014;41:717–723.

[9] Varga JrE, Antal M, Major L, Kiscsatári R, Braunitzer G, Piffkó J. Guidance means accuracy: A randomized clinical trial on freehand versus guided dental implantation. Clin Oral Implants Res. 2020;31:417–430

[10] Lou F, Rao P, Zhang M, Luo S, Lu S, Xiao J. Accuracy evaluation of partially guided and fully guided templates applied to implant surgery of anterior teeth: A randomized controlled trial. Clin Implant Dent Relat Res. 2021;23:117–130

[11] Wei SM, Li Y, Deng K, Lai HC, Tonetti MS, Shi et al. 2024 JY. Does machine-vision- assisted dynamic navigation improve the accuracy of digitally planned prosthetically guided immediate implant placement? A randomized controlled trial. Clin Oral Implants Res. 2022;33:804–815

[12] Yotpibulwong T, Arunjaroensuk S, Kaboosaya B, et al. Accuracy of implant placement with a combined use of static and dynamic computer-assisted implant surgery in single tooth space: A randomized controlled trial. Clin Oral Implants Res. 2023;34:330–341

[13] Shi JY, Liu BL, Wu XY, et al. Improved positional accuracy of dental implant placement using a haptic and machine-vision-controlled collaborative surgery robot: A pilot randomized controlled trial. J Clin Periodontol. 2024;51:24–32

[14] Yang F, Chen J, Cao R, et al. Comparative analysis of dental implant placement accuracy: semi-active robotic versus free-hand techniques: a randomized controlled clinical trial. Clin Implant Dent Relat Res 2024:1–13

[15] Shen P, Zhao J, Fan L, Qiu H, Xu W, Wang Y, et al. Accuracy evaluation of computer-designed surgical guide template in oral implantology. J Craniomaxillofac Surg. 2015;43:2189–2194

[16] Jaemsuwan S, Arunjaroensuk S, Kaboosaya B, Subbalekha K, Mattheos N, Pimkhaokham A. Comparison of the accuracy of implant position among freehand implant placement, static and dynamic computer-assisted implant surgery in fully edentulous patients: a non-randomized prospective study. Int J Oral Maxillofac Surg. 2023;52:264–271

[17] Lorwicheanrung J, Mahardawi B, Arunjaroensuk S, Kaboosaya B, Mattheos N, Pimkhaokham A. The accuracy of implant placement using a combination of static and dynamic computer-assisted implant surgery in fully edentulous arches: A prospective controlled clinical study. Clin Oral Implants Res. 2024;35:841–853

[18] Younis H, Lv C, Xu B, et al. Accuracy of dynamic navigation compared to static surgical guides and the freehand approach in implant placement: a prospective clinical study. Head Face Med. 2024;20:30

[19] Alevizakos V, Mitov G, Stoetzer M, von See C. A retrospective study of the accuracy of template-guided versus freehand implant placement: a non radiologic method. Oral Surg Oral Med Oral Pathol Oral Radiol. 2019;128:220–226

[20] Geng N, Ren J, Zhang C, Zhou T, Feng C, Chen S. Immediate implant placement in the posterior mandibular region was assisted by dynamic real- time navigation: A retrospective study. BMC Oral Health. 2024;24:208

[21] Li J, Dai M, Wang S, Zhang X, Fan Q, Chen L. Accuracy of immediate anterior implantation using static and robotic computer-assisted implant surgery: a retrospective study. J Dent. 2024;148:105218

[22] Stefanelli LV, Mandelaris GA, Franchina A, Di Nardo D, Galli M, Pagliarulo M, Testarelli L, Di Carlo S, Gambarini G. Accuracy evaluation of 14 maxillary full arch implant treatments performed with da vinci bridge: a case series. materials (basel). 2020 Jun 22;13(12):2806. doi: 10.3390/ma13122806. PMID: 32580340; PMCID: PMC7344455.

[23] Wu D, Zhou L, Yang et al. 2024 J, Zhang B, Lin Y, Chen J, Huang W, Chen Y. Accuracy of dynamic navigation compared to static surgical guide for dental implant placement. Int J Implant Dent. 2020 Nov 24;6(1):78. doi: 10.1186/s40729-020-00272-0. PMID: 33230662; PMCID: PMC7683639.

[24] Liu Q, Liu Y, Chen D, Wu X, Huang R, Liu R, Chen Z, Chen Z. Placement accuracy and primary stability of implants in the esthetic zone using dynamic and static computer-assisted navigation: a retrospective case-control study. J Prosthet Dent. 2024 Mar;131(3):427-435. doi: 10.1016/j.prosdent.2022.11.005. Epub 2022 Dec 5. PMID: 36473750.

[25] Feng Y, Su Z, Mo A, Yang et al. 2024 X. Comparison of the accuracy of immediate implant placement using static and dynamic computer-assisted implant system in the esthetic zone of the maxilla: a prospective study. Int J Implant Dent. 2022 Dec 13;8(1):65. doi: 10.1186/s40729-022-00464-w. PMID: 36512162; PMCID: PMC9747989.

[26] Jorba-García A, Bara-Casaus JJ, Camps-Font O, Sánchez-Garcés MÁ, Figueiredo R, Valmaseda-Castellón E. Accuracy of dental implant placement with or without the use of a dynamic navigation assisted system: a randomized clinical trial. Clin Oral Implants Res. 2023 May;34(5):438-449. doi: 10.1111/clr.14050. Epub 2023 Feb 26. PMID: 36794798.

[27] Yimarj P, Subbalekha K, Dhanesuan K, Siriwatana K, Mattheos N, Pimkhaokham A. Comparison of the accuracy of implant position for two-implants supported fixed dental prosthesis using static and dynamic computer-assisted implant surgery: a randomized controlled clinical trial. Clin Implant Dent Relat Res. 2020 Dec;22(6):672-678. doi: 10.1111/cid.12949. Epub 2020 Sep 17. PMID: 32939934.

[28] Kaewsiri D, Panmekiate S, Subbalekha K, Mattheos N, Pimkhaokham A. The accuracy of static vs. dynamic computer-assisted implant surgery in single tooth space: a randomized controlled trial. Clin Oral Implants Res. 2019 Jun;30(6):505-514. doi: 10.1111/clr.13435. Epub 2019 May 7. PMID: 31060099.

[29] Magrin GL, Rafael SNF, Passoni BB, Magini RS, Benfatti CAM, Gruber R, Peruzzo DC. Clinical and tomographic comparison of dental implants placed by guided virtual surgery versus conventional technique: a split-mouth randomized clinical trial. J Clin Periodontol. 2020 Jan;47(1):120-128. doi: 10.1111/jcpe.13211. Epub 2019 Nov 14. PMID: 31628873.

[30] Schneider D, Sancho-Puchades M, Mir-Marí J, Mühlemann S, Jung R, Hämmerle C. A randomized controlled clinical trial comparing conventional and computer-assisted implant planning and placement in partially edentulous patients. Part 4: Accuracy of implant placement. int j periodontics restorative dent. 2019 Jul/Aug;39(4):e111-e122. doi: 10.11607/prd.4147. PMID: 31226190.

[31] Younes F, Cosyn J, De Bruyckere T, Cleymaet R, Bouckaert E, Eghbali A. A randomized controlled study on the accuracy of free-handed, pilot-drill guided and fully guided implant surgery in partially edentulous patients. J Clin Periodontol. 2018 Jun;45(6):721-732. doi: 10.1111/jcpe.12897. Epub 2018 May 10. PMID: 29608793.

[32] Block MS, Emery RW, Cullum DR, Sheikh A. Implant placement is more accurate using dynamic navigation. j oral maxillofac surg. 2017 Jul;75(7):1377-1386. doi: 10.1016/j.joms.2017.02.026. Epub 2017 Mar 14. PMID: 28384461.

[33] Geng et al. 2024 W, Liu C, Su Y, Li J, Zhou Y. Accuracy of different types of computer-aided design/computer-aided manufacturing surgical guides for dental implant placement. Int J Clin Exp Med. 2015 Jun 15;8(6):8442-9. PMID: 26309497; PMCID: PMC4538014.

[34] Cassetta M, Giansanti M, Di Mambro A, Stefanelli LV. Accuracy of positioning of implants inserted using a mucosa-supported stereolithographic surgical guide in the edentulous maxilla and mandible. Int J Oral Maxillofac Implants. 2014 Sep-Oct;29(5):1071-8. doi: 10.11607/jomi.3329. PMID: 25216132.

[35] Cassetta M, Stefanelli LV, Giansanti M, Di Mambro A, Calasso S. Depth deviation and occurrence of early surgical complications or unexpected events using a single stereolithographic surgi-guide. Int J Oral Maxillofac Surg. 2011 Dec;40(12):1377-87. doi: 10.1016/j.ijom.2011.09.009. Epub 2011 Oct 15. PMID: 22001378.

[36] Cristache CM, Burlibasa M, Tudor I, Totu EE, Di Francesco F, Moraru L. Accuracy, labor-time and patient-reported outcomes with partially versus fully digital workflow for flapless guided dental implants insertion-a randomized clinical trial with one-year follow-up. J Clin Med. 2021 Mar 6;10(5):1102. doi: 10.3390/jcm10051102. PMID: 33800946; PMCID: PMC7961841.

[37] Derksen W, Wismeijer D, Flügge T, Hassan B, Tahmaseb A. The accuracy of computer-guided implant surgery with tooth-supported, digitally designed drill guides based on CBCT and intraoral scanning. A prospective cohort study. Clin Oral Implants Res. 2019 Oct;30(10):1005-1015. doi: 10.1111/clr.13514. Epub 2019 Sep 9. PMID: 31330566.

[38] D'haese J, Van De Velde T, Elaut L, De Bruyn H. A prospective study on the accuracy of mucosally supported stereolithographic surgical guides in fully edentulous maxillae. Clin Implant Dent Relat Res. 2012 Apr;14(2):293-303. doi: 10.1111/j.1708-8208.2009.00255.x. Epub 2009 Nov 10. PMID: 19906267.

[39] Di Giacomo GA, da Silva JV, da Silva AM, Paschoal GH, Cury PR, Szarf G. Accuracy and complications of computer-designed selective laser sintering surgical guides for flapless dental implant placement and immediate definitive prosthesis installation. J Periodontol. 2012 Apr;83(4):410-9. doi: 10.1902/jop.2011.110115. Epub 2011 Aug 5. PMID: 21819249.

[40] Ersoy AE, Turkyilmaz I, Ozan O, McGlumphy EA. Reliability of implant placement with stereolithographic surgical guides generated from computed tomography: clinical data from 94 implants. J Periodontol. 2008 Aug;79(8):1339-45. doi: 10.1902/jop.2008.080059. PMID: 18672982.

[41] Fürhauser R, Mailath-Pokorny G, Haas R, Busenlechner D, Watzek G, Pommer B. Esthetics of flapless single-tooth implants in the anterior maxilla using guided surgery: association of three-dimensional accuracy and pink esthetic score. Clin Implant Dent Relat Res. 2015 Oct;17 Suppl 2:e427-33. doi: 10.1111/cid.12264. Epub 2014 Oct 27. PMID: 25346154.

[42] Gelpi F, Modena N, Poscolere A, Bernardello F, Torroni L, De Santis D. Accuracy of computer-guided implantology with pilot drill surgical guide: retrospective 3d radiologic investigation in partially edentulous patients. Medicina (Kaunas). 2023 Apr 10;59(4):738. doi: 10.3390/medicina59040738. PMID: 37109696; PMCID: PMC10142633.

[43] Hanozin B, Li Manni L, LecLou et al. 2021x G, Bacevic M, Lambert F. Digital vs. conventional workflow for one-abutment one-time immediate restoration in the esthetic zone: a randomized controlled trial. Int J Implant Dent. 2022 Feb 7;8(1):7. doi: 10.1186/s40729-022-00406-6. PMID: 35129763; PMCID: PMC8821739.

[44] Kiatkroekkrai P, Takolpuckdee C, Subbalekha K, Mattheos N, Pimkhaokham A. Accuracy of implant position when placed using static computer-assisted implant surgical guides manufactured with two different optical scanning techniques: a randomized clinical trial. Int J Oral Maxillofac Surg. 2020 Mar;49(3):377-383. doi: 10.1016/j.ijom.2019.08.019. Epub 2019 Sep 20. PMID: 31543382.

[45] Kraft B, Frizzera F, de Freitas RM, de Oliveira GJLP, Marcantonio Junior E. Impact of fully or partially guided surgery on the position of single implants immediately placed in maxillary incisor sockets: A randomized controlled clinical trial. Clin Implant Dent Relat Res. 2020 Oct;22(5):631-637. doi: 10.1111/cid.12941. Epub 2020 Sep 1. PMID: 32875722.

[46] Lee JH, Park JM, Kim SM, Kim MJ, Lee JH, Kim MJ. An assessment of template-guided implant surgery in terms of accuracy and related factors. J Adv Prosthodont. 2013 Nov;5(4):440-7. doi: 10.4047/jap.2013.5.4.440. Epub 2013 Nov 28. PMID: 24353883; PMCID: PMC3865200.

[47] Ngamprasertkit C, Aunmeungthong W, Khongkhunthian P. The implant position accuracy between using only surgical drill guide and surgical drill guide with implant guide in fully digital workflow: a randomized clinical trial. Oral Maxillofac Surg. 2022 Jun;26(2):229-237. doi: 10.1007/s10006-021-00975-7. Epub 2021 Jun 23. PMID: 34164754.

[48] Nickenig HJ, Wichmann M, Hamel J, Schlegel KA, Eitner S. Evaluation of the difference in accuracy between implant placement by virtual planning data and surgical guide templates versus the conventional free-hand method - a combined in vivo - in vitro technique using cone-beam CT (Part II). J Craniomaxillofac Surg. 2010 Oct;38(7):488-93. doi: 10.1016/j.jcms.2009.10.023. Epub 2009 Nov 24. PMID: 19939691.

[49] Orban K, Varga et al. 2020 E Jr, Windisch P, Braunitzer G, Molnar B. Accuracy of half-guided implant placement with machine-driven or manual insertion: a prospective, randomized clinical study. Clin Oral Investig. 2022 Jan;26(1):1035-1043. doi: 10.1007/s00784-021-04087-0. Epub 2021 Aug 16. PMID: 34401946; PMCID: PMC8791874.

[50] Ozan O, Turkyilmaz I, Ersoy AE, McGlumphy EA, Rosenstiel SF. Clinical accuracy of 3 different types of computed tomography-derived stereolithographic surgical guides in implant placement. J Oral Maxillofac Surg. 2009 Feb;67(2):394-401. doi: 10.1016/j.joms.2008.09.033. PMID: 19138616.

[51] Pellegrino G, Taraschi V, Andrea Z, Ferri A, Marchetti C. Dynamic navigation: a prospective clinical trial to evaluate the accuracy of implant placement. Int J Comput Dent. 2019;22(2):139-147. PMID: 31134220.

[52] Schnutenhaus S, Edelmann C, Rudolph H, Luthardt RG. Retrospective study to determine the accuracy of template-guided implant placement using a novel nonradiologic evaluation method. Oral Surg Oral Med Oral Pathol Oral Radiol. 2016 Apr;121(4):e72-9. doi: 10.1016/j.oooo.2015.12.012. Epub 2016 Jan 4. PMID: 26972545.

[53] Stübinger S, Buitrago-Tellez C, Cantelmi G. Deviations between placed and planned implant positions: an accuracy pilot study of skeletally supported stereolithographic surgical templates. Clin Implant Dent Relat Res. 2014 Aug;16(4):540-51. doi: 10.1111/cid.12019. Epub 2012 Nov 21. PMID: 23167722.

[54] Wei SM, Shi JY, Qiao SC, Zhang X, Lai HC, Zhang XM. Accuracy and primary stability of tapered or straight implants placed into fresh extraction socket using dynamic navigation: a randomized controlled clinical trial. Clin Oral Investig. 2022 Mar;26(3):2733-2741. doi: 10.1007/s00784-021-04247-2. Epub 2021 Nov 19. PMID: 34797431.

[55] Huang L, Liu L, Yang et al. 2024 S, Khadka P, Zhang S. Evaluation of the accuracy of implant placement by using implant positional guide versus freehand: a prospective clinical study. Int J Implant Dent. 2023 Dec 1;9(1):45. doi: 10.1186/s40729-023-00512-z. PMID: 38036932; PMCID: PMC10689697.

[56] Han X, Qi C, Guo P, Zhang S, Xu Y, Lv G, Li Y, Li C. Whole-process digitalization-assisted immediate implant placement and immediate restoration in the aesthetic zone: a prospective study. Med Sci Monit. 2021 Aug 24;27:e931544. doi: 10.12659/MSM.931544. PMID: 34428195; PMCID: PMC8400573.

[57] Zhao N, Du L, Lv C, Liang J, He L, Zhou Q. Accuracy analysis of robotic-assisted immediate implant placement: a retrospective case series. J Dent. 2024 Jul;146:105035. doi: 10.1016/j.jdent.2024.105035. Epub 2024 May 9. PMID: 38734299.

[58] Wu Y, Zou S, Lv P, Wang X. Accuracy of an autonomous dental implant robotic system in dental implant surgery. J Prosthet Dent. 2025 Mar;133(3):764-770. doi: 10.1016/j.prosdent.2024.07.020. Epub 2024 Aug 13. PMID: 39142994.

[59] Wang M, Liu F, Yu T, Zhan Y, Ma F, Rausch-Fan X. Accuracy of an autonomous dental implant robotic system in partial edentulism: A pilot clinical study. Clin Oral Investig. 2024 Jun 19;28(7):385. doi: 10.1007/s00784-024-05780-6. PMID: 38890168.

[60] Xie R, Liu Y, Wei et al. 2022 H, Zhang T, Bai S, Zhao Y. Clinical evaluation of autonomous robotic-assisted full-arch implant surgery: A 1-year prospective clinical study. Clin Oral Implants Res. 2024 Apr;35(4):443-453. doi: 10.1111/clr.14243. Epub 2024 Feb 6. PMID: 38318691.

[61] Neugarten JM. Accuracy and precision of haptic robotic-guided implant surgery in a large consecutive series. Int J Oral Maxillofac Implants. 2024 Feb 27;39(1):99-106. doi: 10.11607/jomi.10468. PMID: 37939239.

[62] Zhang S, Cai Q, Chen W, Lin Y, Gao Y, Wu D, Chen J. Accuracy of implant placement via dynamic navigation and autonomous robotic computer-assisted implant surgery methods: a retrospective study. Clin Oral Implants Res. 2024 Feb;35(2):220-229. doi: 10.1111/clr.14216. Epub 2023 Nov 30. PMID: 38033198.

[63] Li P, Chen J, Li A, Luo K, Xu S, Yang et al. 2024 S. Accuracy of autonomous robotic surgery for dental implant placement in fully edentulous patients: a retrospective case series study. Clin Oral Implants Res. 2023 Dec;34(12):1428-1437. doi: 10.1111/clr.14188. Epub 2023 Oct 19. PMID: 37855331.

[64] Wang W, Xu H, Mei D, Zhou C, Li X, Han Z, Zhou X, Li X, Zhao B. Accuracy of the Yakebot dental implant robotic system versus fully guided static computer-assisted implant surgery template in edentulous jaw implantation: a preliminary clinical study. Clin Implant Dent Relat Res. 2024 Apr;26(2):309-316. doi: 10.1111/cid.13278. Epub 2023 Sep 20. PMID: 37728030.

[65] Qiao SC, Wu XY, Shi et al. 2024 JY, Tonetti MS, Lai HC. Accuracy and safety of a haptic operated and machine vision controlled collaborative robot for dental implant placement: a translational study. Clin Oral Implants Res. 2023 Aug;34(8):839-849. doi: 10.1111/clr.14112. Epub 2023 Jun 12. PMID: 37309242.

[66] Chen W, Al-Taezi KA, Chu CH, Shen et al. 2015 Y, Wu J, Cai K, Chen P, Tang C. Accuracy of dental implant placement with a robotic system in partially edentulous patients: a prospective, single-arm clinical trial. Clin Oral Implants Res. 2023 Jul;34(7):707-718. doi: 10.1111/clr.14083. Epub 2023 May 11. PMID: 37167364.

[67] Jia S, Wang G, Zhao Y, Wang X. Accuracy of an autonomous dental implant robotic system versus static guide-assisted implant surgery: A retrospective clinical study. J Prosthet Dent. 2025 Mar;133(3):771-779. doi: 10.1016/j.prosdent.2023.04.027. Epub 2023 Jun 7. PMID: 37291043.

[68] Yang et al. 2024 S, Chen J, Li A, Deng K, Li P, Xu S. Accuracy of autonomous robotic surgery for single-tooth implant placement: A case series. J Dent. 2023 May;132:104451. doi: 10.1016/j.jdent.2023.104451. Epub 2023 Feb 11. PMID: 36781099.

[69] Bolding SL, Reebye UN. Accuracy of haptic robotic guidance of dental implant surgery for completely edentulous arches. J Prosthet Dent. 2022 Oct;128(4):639-647. doi: 10.1016/j.prosdent.2020.12.048. Epub 2021 Mar 5. PMID: 33678441.
